# Using Social Media for the Prevention of Pediatric Burn Injuries: Pilot Design and Usability Study

**DOI:** 10.2196/23242

**Published:** 2021-07-15

**Authors:** Nikita Batra, Cindy D Colson, Emily C Alberto, Randall S Burd

**Affiliations:** 1 Children's National Hospital Washington, DC United States

**Keywords:** accident prevention, burns, pediatric, public health, social media

## Abstract

**Background:**

Most pediatric burn injuries are preventable. Social media is an effective method for delivering large-scale messaging and may be useful for injury prevention in this domain.

**Objective:**

This study evaluates the feasibility of creating a social media campaign for pediatric burn injury prevention.

**Methods:**

Ad spots containing a headline, short introduction, and video were created and posted on Facebook and Instagram over 4 months. Ad spots were targeted to parents and caregivers of children in our region with the highest number of burn injuries. We assessed the impact of each ad set using ThruPlays, reach, and video plays.

**Results:**

We created 55 ad spots, with an average length of 24.1 (range 10-44) seconds. We reached 26,496 people during the campaign. The total ThruPlays of the 55 ad spots were 14,460 at US $0.19 per ThruPlay. Ad spots related to home safety had a significantly higher daily ThruPlay rate than those related to fire safety (6.5 vs 0.5 per day; *P*<.001).

**Conclusions:**

Social media is a feasible modality for delivering public health messages focused on preventing pediatric burn injuries. Engagement with these ads is influenced by ad presentation and the focus of the underlying injury prevention message.

## Introduction

Burns are a leading cause of injury among children 5 years old and younger in the United States. Approximately 50,000 children were treated in the emergency department for unintentional burns in 2018 [1]. Among all etiologies, scald burns are the most common type sustained by children. Most scald burns are related to spills or contact with hot food, beverages, or tap water, making the kitchen and the bathroom common locations for these injuries in the home [2]. Although most pediatric burns are minor and can be managed in an outpatient setting, children with severe burns require acute and long-term management at a pediatric burn center [3,4]. The medical cost of pediatric burn–related hospitalizations was close to $150 million in 2010 [5]. In addition to the associated cost, burn injuries can cause significant psychological burdens on patients and their families [6]. Because pediatric burn injuries are mostly preventable, outreach programs have successfully increased parental knowledge of possible dangers and reduced hospitalization rates of burn injuries in children [7,8].

Mass media campaigns that use radio, television, newspapers, and the internet have successfully encouraged behaviors that reduce sports-related injuries and injuries from motor vehicle crashes [9]. Social media has emerged as a new platform for health promotion in many areas, including smoking cessation, exercise, and diet [10-12]. In addition to reaching large numbers of people, social media supports information sharing by users, further amplifying its impact. Social media may be more influential than traditional media campaigns because of the interaction between users and the produced content [13,14]. Although utilizing social media for injury prevention is relatively new, initial experience suggests that this approach is feasible and effective for delivering this type of message [15,16].

To address the burden of pediatric burn injuries in our region, we collaborated with a creative marketing company to create a social media advertising campaign targeted at the caregivers of children in our area. This study aimed to assess the feasibility of and potential factors for determining the success of a social media campaign for pediatric burn injuries.

## Methods

### Overview

Children’s National Hospital is a regional pediatric burn center designated by the Maryland Institute for Emergency Medical Services Systems that treats more than 850 burn patients annually in both inpatient and outpatient settings. In 2018, we created the “After the Burn” series to provide burn prevention education and information on treatment and recovery following a pediatric burn injury. The video content was generated from over 35 interdisciplinary interviews conducted at our institution and has been available on YouTube since October 2018 [17]. The video series contains 15 episodes, with 2 focusing on burn injury prevention, 12 on the care of the burned child from injury through recovery, and 1 spotlighting the collaboration between our organization and the District of Columbia Firefighter’s Burn Foundation.

**Figure 1:**
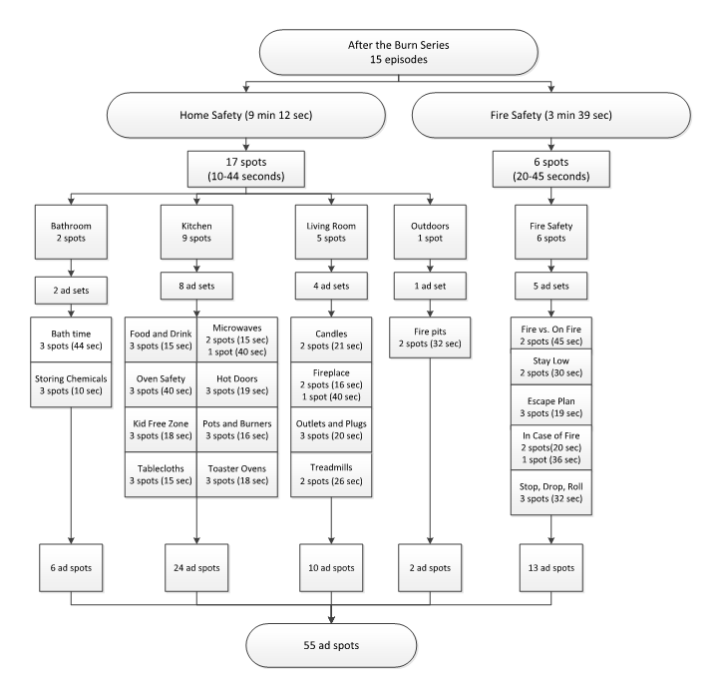
Ad set distributions.

To deliver pediatric burn injury prevention information to a larger audience within our community, we collaborated with Getfused, a creative marketing company from Boston, MA. They generated a burn prevention ad campaign using the two burn injury prevention episodes from the “After the Burn” series [17]. This collaboration enabled us to achieve our goal of distributing content to the prespecified target audience. At Children’s National Hospital, the injury prevention coordinator was responsible for content approval and worked with the Getfused team in preparing and optimizing the ad campaign content. Getfused segmented the “home safety” (runtime: 9 minutes 12 seconds) and “fire safety” (runtime: 3 minutes 39 seconds) videos into spots (ie, short clips from the full-length episode) based on the different locations and mechanisms of burn injuries that could be sustained in the home (Figure 1) [18,19]. The “home safety” episode was divided into 17 spots that included 4 locations where injuries could occur (bathroom, kitchen, living room, and outdoors) [18]. The “fire safety” episode was segmented into 6 spots based on the different actions one must take during a fire [19]. The spots in each category were used to create 20 ad sets. Each spot (10-45 seconds) was combined with a headline and a short introductory text (less than 55 words) to create unique ad spots. Ad sets contained 2 to 3 versions of an ad spot. For most ad sets, the versions included the same spot with either a different headline, introduction, or both. For 3 ad sets (ie, “in case of fire,” “microwaves,” and “fireplace”), the versions had different spots of varying lengths.

The resulting ad spots were formatted for distribution on Facebook’s news and video feeds and Instagram’s feeds and explore pages. Demographic targeting was used to direct the ad sets to parents with a child younger than 18 years of age or household managers or nannies in the District of Columbia who were aged between 24 and 44 years. We ran this campaign on Facebook and Instagram from September 2019 until December 2019.

### Outcome Assessment

We assessed the impact of each ad set using metrics from Facebook Business Manager, including ThruPlays, reach, and video plays. Based on the definition from Facebook, a ThruPlay is the number of times a spot is played to completion or for at least 15 seconds. For spots longer than 15 seconds, a ThruPlay is counted when at least 15 unique seconds of the spot are played. If the spot is shorter than 15 seconds, a ThruPlay is counted when the spot is played to completion [20]. Reach is defined as the number of people who saw an ad spot at least once on their social media feed during a campaign [21]. Reach is not counted when an ad spot is viewed more than once by the same person. A video play is defined as the number of times a spot starts to play, excluding replays (ie, when a spot is paused and then started again). A video play can occur when a spot plays automatically or when a spot is clicked on to play [20]. We grouped video plays into categories based on the percentage of the spot length (25%, 50%, and 100%) played, including plays that skipped to parts of the videos within these percentages of play [20]. Cost per result is a metric used for ad campaigns that report the amount spent per result, where the result is defined based on the objective of the campaign [21]. For this study, we used ThruPlays because they best reflect the attention focused on the intended video messaging.

We used aggregate data from Facebook and Instagram for our analyses. Aided by these performance metrics, Getfused team members modified the ad campaign biweekly to generate the highest possible engagement within the target audience. The injury prevention coordinator from Children’s National Hospital received bi-weekly updates on campaign statistics and provided input during each change. Changes included modification of ad spot headlines or introductions within an ad set. Ad spot versions were removed from the campaign when performance indicators showed limited views. The runtime reflects the average number of days that an ad spot version in an ad set was run. Performance metrics were averaged among different versions of the ad spot within each ad set. We also summed the performance metrics across all ad spots to generate the overall campaign performance. We conducted comparisons between ThruPlay rates using the Poisson exact test that evaluates the null hypothesis about the difference between rates. Ad spots derived from the “home safety” episode were compared to ad spots derived from the “fire safety” episode. Among ad spots derived from the “home safety” episode, those in the “kitchen” category were compared to all other ad spots. We made this comparison because most pediatric burn injuries at home occur in the kitchen [22,23]. The “storing chemicals” ad set was the only ad set that included ad spots less than 15 seconds. Because a ThruPlay is counted when ad spots less than 15 seconds long are played to completion, we evaluated daily ThruPlay rates with and without the inclusion of this ad set to assess the impact of short duration videos on rate differences. A multiple linear regression was performed to evaluate the association of ad spot category with ThruPlay rate, controlling for video duration. We defined significance at *P*<.05.

## Results

We created 55 ad spots, with an average length of 24.1 (range 10-44) seconds. The runtime of the ad spots averaged 44.7 (range 3-109) days (Table 1). We reached 26,496 unique people during the campaign. These individuals collectively executed 14,460 ThruPlays of the 55 ad spots (average 239 ThruPlays per ad spot). The “storing chemicals” ad set had the highest average of ThruPlays (36.1 per day). Among ad sets with durations longer than 15 seconds, the “oven safety” ad set had the highest average of ThruPlays (7.3 per day), while the “candles” ad set had the lowest average of ThruPlays (0.1 per day). Our advertising budget for the campaign was US $2750, which translated to US $0.19 per ThruPlay based on the number of individuals reached (cost per result). The costs of content preparation, development of a campaign strategy, and campaign optimization were separately funded.

Ad spots derived from the “home safety” episode had a higher daily ThruPlay rate than ad spots derived from the “fire safety” episode (6.5 vs 0.5 per day; *P*<.001). The difference between these groups remained significant when the “storing chemicals” ad set was excluded (2.5 vs 0.5 per day; *P*<.001). Among all the ad spots derived from the “home safety” episode, ad spots in the “kitchen” category had a lower ThruPlay rate than other ad sets (3.4 vs 10.6 per day; *P*<.001). When the “storing chemicals” (short duration) ad set was excluded, this difference was reversed. ad spots in the “kitchen” category had a higher daily ThruPlay rate than other ad spots (3.4 vs 1.1 per day; *P*<.001).

Among ad spots that included videos longer than 15 seconds (ie, excluding “storing chemicals” ad spots), the “home safety” episodes had a higher ThruPlay rate than “fire safety” episodes (an increase of 1.8 ThruPlays per day; *P*=.02), controlling for the duration of the video. The total video plays of the 55 ad spots were 293,109. Although only 4.1% (n=12,090) of initiated views were played to completion, 24.2% (n=27,178) of initiated watches were played for more than 50% (range 5-22.5 seconds) of the video length. Consistent with its short length, the “storing chemicals” ad set had the most video plays to completion per day (35 video plays per day; Table 2).

Observational analysis, obtained during ad modifications by Getfused, showed two features of the ad spot that improved performance: (1) introductory text with specific objects that caused burns rather than the general locations where burns occurred, and (2) headlines starting with the word “prevent” or the specific household item that could cause burns. Examples of headlines in these two categories include “chemicals can cause burn risks to your child” and “prevent chemical burns from happening to your child,” respectively.

**Table 1:** Ad set summaries.

Ad sets		Runtime (days), average	Reach (people per day), average	ThruPlays (views per day), average
**Home safety: bathroom**				
	Bath time	56	30	2
	Storing chemicals	67	144	36
**Home safety: kitchen**				
	Food and drinks	30	35	3
	Microwaves (15 sec^a^)	49	39	3
	Microwaves (40 sec)	3	2	0
	Oven safety	56	64	7
	Hot doors	55	48	3
	Kid free zones	51	70	5
	Pots and burners	55	22	1
	Tablecloths	45	27	2
	Toaster ovens	50	34	2
**Home safety: living room**				
	Candles	27	4	0
	Fireplaces (16 sec)	42	16	1
	Fireplaces (40 sec)	55	12	1
	Outlets and plugs	38	11	1
	Treadmills	55	17	1
**Home safety: outdoors**				
	Firepits	42	28	2
**Fire safety**				
	Escape plan	32	8	0
	Fire vs. on fire	54	16	1
	In case of fire (20 sec)	17	10	0
	In case of fire (36 sec)	11	7	0
	Stay low and go	54	11	0
	Stop, drop and roll	40	9	0

^a^sec: seconds

**Table 2:** Ad set video plays.

Ad Sets			Video plays at 25% (plays per day), average		Video plays at 50% (plays per day), average		Video plays at 100% (plays per day), average		Total video plays (plays per day), average
**Home safety: bathroom**									
	Bath time	2		1		1		38	
	Storing chemicals	167		83		35		712	
**Home safety: kitchen**									
	Food and drinks	7		4		2		42	
	Microwaves (15 sec^a^)	7		4		2		51	
	Microwaves (40 sec)	0		0		0		2	
	Oven safety	19		10		5		151	
	Hot doors	7		4		2		62	
	Kid free zones	14		7		3		97	
	Pots and burners	4		2		1		26	
	Tablecloths	5		3		1		34	
	Toaster ovens	5		3		2		38	
**Home safety: living room**									
	Candles	0		0		0		4	
	Fireplaces (16 sec)	2		1		1		17	
	Fireplaces (40 sec)	1		1		0		14	
	Outlets and plugs	1		1		1		13	
	Treadmills	2		1		0		19	
**Home safety: outdoors**									
	Firepits	3		1		1		35	
**Fire safety**									
	Escape plan	1		1		0		7	
	Fire vs. on fire	1		0		0		17	
	In case of fire (20 sec)	1		1		0		10	
	In case of fire (36 sec)	0		0		0		6	
	Stay low and go	1		0		0		10	
	Stop, drop and roll	0		0		0		10	

^a^sec: seconds

## Discussion

### Principal Results and Comparison With Prior Work

Social media has grown in popularity over the last decade, with Facebook reporting more than 1.4 billion daily adult users and Instagram more than 500 million daily users [24,25]. Given the number of people using social media, organizations interested in health care promotion have evaluated this modality to encourage healthy behaviors. Examples of health promotion campaigns using social media include those that promote smoking cessation, diabetes management, and obesity prevention [26-28].

Although social media may have a similar value in injury prevention, few studies have evaluated its use in this domain. For instance, an Instagram account posting messages promoting adolescent seat belt use over 3 months was introduced at a high school health fair. Using “likes” as a metric of a successful campaign, posts presented positively or those involving celebrities or humor received the most attention [15]. In another study, Facebook advertising was used to disseminate information about fall prevention in an elderly population, reaching more than 140,000 people in British Columbia, Canada. Among the people the ad reached, 3% engaged with the content through link clicks, reactions, comments, and shares [29]. These studies show the feasibility of a social media injury prevention campaign and initial evidence of its potential impact [15,29].

The use of video advertising contributed to the success of our campaign. Before designing the campaign, the “After the Burn” series was created to provide clear and detailed explanations of each step in the recovery process following a pediatric burn injury. The goal of this video series was to decrease parental stress, improve the quality of life for the child, and improve postinjury medical outcomes [17]. We posted these on YouTube, allowing access to individuals in the general population who might be seeking burn injury prevention information online. We also directed hospitalized patients with burn injuries and their parents or guardians to these videos in their discharge instructions. The purpose of this campaign was to expand the reach of the injury prevention videos using social media as a platform for targeting populations in our region.

Traditional television, radio, or print campaigns can often reach large audiences. By targeting specific populations, social media campaigns have an advantage over these traditional campaigns [30]. Specific audience targeting also led to the success of our ad campaign. Our campaign directed content to individuals for whom pediatric burn injury prevention was most relevant: the parents, guardians, and caretakers of children. As a result, the number of individuals reached was significantly higher than would be reached at a typical health fair [31-33]. In addition, the cost per result of the ad campaign was lower than the industry standard of $1 per ThruPlay, supporting the cost efficiency of this campaign. We selected ThruPlays as the outcome metric for our campaign, aligning with our initial objective to promote video play among reached individuals. The use of ThruPlays for this purpose assumes that watching 15 seconds of a video delivers the ad’s main message sufficiently. Facebook research supports this assumption, showing that even plays of less than 15 seconds can increase ad recall, brand awareness, and purchase intent [34].

Video length is a factor that can be modified to influence user engagement. In this study, the “storing chemicals” ad set had the highest average ThruPlay rate, consistent with having videos with the shortest duration. According to Facebook video statistics, engagement with video content occurs with a watch duration longer than 10 seconds [35]. Many of the videos in our ad campaign meet this benchmark. However, video duration was not associated with the ThruPlay rate for ad spots longer than 15 seconds. This observation suggests the length of the video, observable in the progress bar, may not discourage individuals from watching for longer durations.

Our observation aligns with evidence that viewer engagement remains constant for videos up to 2 minutes long, after which engagement declines [36]. Although the standardization of video length would allow for the evaluation of the impact of video length on user engagement, the differing content of each ad makes this type of standardization challenging. In future evaluations of the impact of social media campaigns, modifying the standard Facebook definition of a ThruPlay will better assess the impact of video duration on engagement.

Although up to 90% of pediatric burns occur in the home, many parents are unaware of the risks that can lead to burn injuries in this setting [23,37-39]. Parents are also often unaware that pediatric burn injuries in the home occur most often in the kitchen [22,23,40]. After excluding the short duration “storing chemicals” ad set, ad spots in the kitchen category had a higher ThruPlay rate than other ad spots derived from the “home safety” episode. Two factors may account for these differences: (1) interest may be higher for ad spots that are more relevant to everyday life in the home, such as kitchen-related activities, than less frequent fire safety events, and (2) individuals may also have established knowledge on fire safety from previous successful media campaigns, increasing the novelty of the “home safety” ad spots [41-43]. Establishing the reasons for the differences in the ThruPlay rates is needed to optimize the impact of social media campaigns.

Based on an assessment of available ad spot metrics used by Getfused, we observed that modifications in the headlines and introductory text improved ad spot performance for pediatric burn injury prevention. Previous social media campaigns have established the impact of front-end content on ad reach and engagement [15,44]. Modifying front-end factors, such as the introductory text, can increase positive engagement with ads by as much as 2-fold in social media campaigns [15,44]. Several approaches can be used to evaluate the optimal ad spot presentation, including A/B testing, multivariate testing, and multipage testing. Our campaign used video-sharing as its medium because of the content available in this format. Several other advertising media can be used to create ad campaigns, including photo sharing, text sharing, and microblogging.

### Limitations

Our study had several limitations. First, although we identified several parameters for optimizing a burn injury prevention campaign using social media, we did not evaluate whether this campaign changed the incidence of burn injuries in our region. This pilot study showed the potential role of a social media campaign for sharing burn injury prevention information and the features that can be modified to optimize its reach. We are developing a follow-up campaign optimized based on this study in which the impact on burn incidence will be measured. Second, although data from our burn database shows that young children are more likely to sustain scald burns, we could not target parents based on the age of their children. As a result, we could not evaluate whether the parents of younger children were more likely to watch the videos. Third, understanding key performance metrics before the campaign launch would have allowed us to obtain more specific reports during the optimization phases. The creative marketing company made several changes in the presentation of the ad spots during predetermined optimization periods using standard industry practices. It is common to use this “on the fly” approach without a defined feedback loop or set evaluation metrics [45]. These changes were made rapidly, precluding objective evaluation of each (eg, headline or introductory text) in this pilot. Finally, we used ThruPlays as the primary metric for viewer engagement. A follow-up social media campaign will include an assessment of which metrics most influence the impact of each ad.

### Conclusions

Pediatric burn injuries can have significant long-lasting physical and psychological impacts on patients and their families. Because these injuries are mostly preventable through actions taken by parents and caretakers, increasing awareness of these injuries and the mechanisms will reduce the associated health burden. Although mass media campaigns have reduced the risk of injury by other means, using social media specifically for this purpose has only recently received attention. Although it is challenging to define benchmarks for success given the many factors related to burn injury in children, our burn prevention campaign exceeded published standards as measured by ThruPlays, total campaign reach, and the cost per result. Successful injury prevention ad campaigns should ideally use video (specifically short videos), use audience targeting, implement predetermined performance metrics, and apply optimization methods to improve performance.
